# Supplementary material for: Honeybee gut bacterial strain improved survival and gut microbiota homeostasis in Apis mellifera exposed in vivo to clothianidin
Source: Microbiol Spectr. 2024 Aug 27;12(10):e00578-24. doi: 10.1128/spectrum.00578-24 (PMC11448422; doi:10.1128/spectrum.00578-24)
Supplement: Supplemental material — Table S1 to S5. [file spectrum.00578-24-s0001.pdf]

Honey bee gut bacterial strain improved survival and gut microbiota homeostasis in *Apis mellifera* exposed *in vivo* to clothianidin

Sarah El Khoury [1,2,1a], Jeff Gauthier [1,2], Pierre Luc Mercier [1,2], Stéphane Moïse [3], Pierre Giovenazzo [2] and Nicolas Derome [1,2]*

[1] Université Laval, Institut de Biologie Intégrative et des Systèmes (IBIS), Québec, Canada

[2] Université Laval, Département de Biologie, Québec, Canada

[3] INRS, Institut National de la Recherche Scientifique, Québec, Canada

Supplementary material

Table S1. Variability between experimental group was calculated with a Cox Proportional Hazard model (Coxme (mixed effects Cox Model with the factor "cage" as random effect)). Significant differences were calculated using a multiple comparison post-CoxME. P-values were adjusted with the Tukey test. Significant p-values < 0.05 "*"; < 0.01 "**". Empty space in the table is not significative.

	**Pesticide Control**	***Enterobacter***	***Enterobacter* + Pesticide**	***Pantoea***	***Pantoea* + Pesticide**
**Sugar Control**	*	-	-	-	-
**Pesticide Control**		-	-	-	**
Enterobacter			-	-	-
***Enterobacter* + Pesticide**					*
Pantoea					-

[1a] Department of Integrative Biology, University of California, Berkeley, CA, USA

Table S2. Number of ASVs at the genus level in the microbial network depending on each honeybee gut section (midgut, ileum and rectum) and each experimental group.

**Experimental group**	**Gut section**		
	**Midgut**	**Ileum**	**Rectum**
**Sugar Control (0 ppb)**	38	66	38
**Pesticide Control (0.1 ppb)**	37	49	32
***Enterobacter* + (0.1 ppb)**	35	36	33
***Pantoea* + (0.1 ppb)**	26	46	31

Table S3. Number of positive (+) and/or negative (-) interactions at the genus level in the microbial network depending on each honeybee gut section (midgut, ileum and rectum) and each experimental group.

**Experimental group**	(+)			(-)		
	**Midgut**	**Ileum**	**Rectum**	**Midgut**	**Ileum**	**Rectum**
**Sugar Control (0 ppb)**	68	98	38	0	54	13
**Pesticide Control (0.1 ppb)**	39	97	137	3	3	0

Enterobacter **+ (0.1 ppb)**	144	30	50	0	7	4
Pantoea **+ (0.1 ppb)**	34	40	33	2	20	13

Table S4. Network metrics for gut microbiome taxa in rectum exposed to 0.1 ppb clothianidin (Pesticide control group). CC = Closeness centrality, DG = Degree, NC = Neighborhood Connectivity.

Taxa name (spp.)	ASV classification	CC	DG	NC	ASV activity
Lactobacillus	Core	0.65789474	15	13.7333333	107973
Snodgrassella	Core	0.65789474	15	13.7333333	52687
Bartonella	Non-core	0.67567568	15	12.9333333	21701
Gilliamella	Core	0.67567568	16	12.1875	20074
no identification	Unassigned	0.65789474	15	13.7333333	15227
Frischella	Non-core	0.42372881	3	11.3333333	3887
Bombella	Non-core	0.65789474	15	13.7333333	2371
Klebsiella	Low abundant	0.65789474	15	12.3333333	1332
Bifidobacterium	Core	0.58139535	9	11.8888889	1164
Lysinibacillus	Low abundant	0.49019608	6	8.66666667	606
Lachnoclostridium	Low abundant	0.65789474	15	13.7333333	406
Enterobacter	Low abundant	0.65789474	14	13.1428571	379
Parasaccharibacter	Non-core	0.54347826	11	14.0909091	364
Pluralibacter	Low abundant	0.64102564	13	12.6153846	228
Gottschalkia	Low abundant	0.58139535	9	11.8888889	65
Morganella	Low abundant	0.64102564	13	12.6153846	61
Kineothrix	Low abundant	0.65789474	15	13.7333333	53
Shinella	Low abundant	0.36764706	2	4	46
Oceanobacillus	Low abundant	0.48076923	6	10.8333333	18
Bacillus	Low abundant	0.42372881	3	11.3333333	17
Mobilisporobacter	Low abundant	0.51020408	10	13.1	6
Kosakonia	Low abundant	0.48076923	6	8.5	6
Tissierella	Low abundant	0.36764706	2	4	5
Citrobacter	Low abundant	1	1	1	3
Muricomes	Low abundant	1	1	1	3
Ochrobactrum	Low abundant	0.32894737	1	6	3
Shimwellia	Low abundant	0.55555556	12	13.1666667	2

Phyllobacterium	Low abundant	0.55555556	12	13.1666667	2
Erwinia	Low abundant	1	1	1	1
Acinetobacter	Low abundant	1	1	1	1
Dermacoccus	Low abundant	1	1	1	1
Humibacter	Low abundant	1	1	1	1

Table S5. Network metrics for gut microbiome taxa in rectum exposed to 0.1 ppb clothianidin and *Pantoea* (*Pantoea* curative group). CC = Closeness centrality, DG = Degree, NC = Neighborhood Connectivity.

Taxa name (spp.)	ASV classification	CC	DG	NC	ASV activity
Lactobacillus	Core	0.244444444	3	3	174088
Snodgrassella	Core	0.150684932	1	2	53083
Bartonella	Non-core	1	4	3.5	40117
no identification	Unassigned	0.8	3	4	21362
Frischella	Non-core	0.154929577	2	3	14319
Bifidobacterium	Core	1	4	3.5	3918
Bombella	Non-core	0.180327869	3	2.666666667	1943
Parasaccharibacter	Non-core	0.2	1	4	1735
Klebsiella	Low abundant	0.275	3	4	939
Enterobacter	Low abundant	0.323529412	4	4	808
Lachnoclostridium	Low abundant	0.20754717	3	2.333333333	290
Pluralibacter	Low abundant	0.247191011	4	2.75	139
Shinella	Low abundant	0.8	3	4	100
Morganella	Low abundant	0.255813953	3	3.333333333	92
Lysinibacillus	Low abundant	0.211538462	3	3.333333333	46
Kineothrix	Low abundant	0.176	2	2	43
Oceanobacillus	Low abundant	0.268292683	3	4.333333333	36
Ochrobactrum	Low abundant	0.285714286	4	3.75	17
Shimwellia	Low abundant	0.255813953	3	4	15
Erwinia	Low abundant	0.305555556	3	4.666666667	14
Kosakonia	Low abundant	0.323529412	6	3.333333333	11
Saccharibacter	Low abundant	1	4	3.5	10
Citrobacter	Low abundant	0.180327869	3	2.666666667	8
Bacillus	Low abundant	0.278481013	4	3.5	6
Paenibacillus	Low abundant	0.301369863	3	4	3
Lachnotalea	Low abundant	0.205607477	1	3	3
Mobilisporobacter	Low abundant	1	2	2	2

Pseudaminobacter	Low abundant	1	2	2	2
Mesorhizobium	Low abundant	0.305555556	4	3.5	2
Tissierella	Low abundant	0.203703704	2	3	2
Salipiger	Low abundant	1	2	2	1
